# Pre-processing Agilent microarray data

**DOI:** 10.1186/1471-2105-8-142

**Published:** 2007-05-01

**Authors:** Marianna Zahurak, Giovanni Parmigiani, Wayne Yu, Robert B Scharpf, David Berman, Edward Schaeffer, Shabana Shabbeer, Leslie Cope

**Affiliations:** 1 Johns Hopkins University School of Medicine, Oncology Biostatistics, 550 N. Broadway, Baltimore, MD 21205, USA; 2 Johns Hopkins School of Medicine, Baltimore, MD 21231, USA; 3 Johns Hopkins Bloomberg School of Public Health, 615 N. Wolfe St., Room E3034, Baltimore, MD 21205, USA; 4 Johns Hopkins University School of Medicine, 1550 Orleans St., CRB II Room 5.45, Baltimore, MD 21231, USA; 5 Johns Hopkins University School of Medicine, 600 N. Wolfe St., Marburg 145, Baltimore, MD 21287, USA; 6 Johns Hopkins University School of Medicine, 1650 Orleans St., CRB I, Baltimore, MD 21231, USA

## Abstract

**Background:**

Pre-processing methods for two-sample long oligonucleotide arrays, specifically the Agilent technology, have not been extensively studied. The goal of this study is to quantify some of the sources of error that affect measurement of expression using Agilent arrays and to compare Agilent's Feature Extraction software with pre-processing methods that have become the standard for normalization of cDNA arrays. These include log transformation followed by loess normalization with or without background subtraction and often a between array scale normalization procedure. The larger goal is to define best study design and pre-processing practices for Agilent arrays, and we offer some suggestions.

**Results:**

Simple loess normalization without background subtraction produced the lowest variability. However, without background subtraction, fold changes were biased towards zero, particularly at low intensities. ROC analysis of a spike-in experiment showed that differentially expressed genes are most reliably detected when background is not subtracted. Loess normalization and no background subtraction yielded an AUC of 99.7% compared with 88.8% for Agilent processed fold changes. All methods performed well when error was taken into account by t- or z-statistics, AUCs ≥ 99.8%. A substantial proportion of genes showed dye effects, 43% (99%*CI *: 39%, 47%). However, these effects were generally small regardless of the pre-processing method.

**Conclusion:**

Simple loess normalization without background subtraction resulted in low variance fold changes that more reliably ranked gene expression than the other methods. While t-statistics and other measures that take variation into account, including Agilent's z-statistic, can also be used to reliably select differentially expressed genes, fold changes are a standard measure of differential expression for exploratory work, cross platform comparison, and biological interpretation and can not be entirely replaced. Although dye effects are small for most genes, many array features are affected. Therefore, an experimental design that incorporates dye swaps or a common reference could be valuable.

## Background

The latest generation of expression microarray technology offers a snapshot of the entire transcriptome with an accuracy and resolution that was unimaginable just a few years ago. The recent improvements have come from several different directions. On the purely technological front, arrayers, scanners and hybridization chambers have improved significantly in recent years. Laboratory technique has become standardized as microarrays are used more widely. Improved genomic libraries have made it possible to select probes more appropriately and to annotate them with greater accuracy. Advances in array processing and data analysis have made the most of available data within the limitations of current technology. Several microarray technologies are currently available, and new players appear all the time, but a few formats have emerged as leaders in the field. cDNA arrays paved the way more than ten years ago, and remain a widely used platform. Affymetrix introduced the oligonucleotide approach to chip design and quickly became the most widely used expression array format. Agilent, in cooperation with Rosetta, has combined some of the best features of both of these approaches and gained wide spread acceptance as a result.

In the two-color cDNA platform, mRNA from two samples is reverse-transcribed to cDNA, labeled with fluorescent dye, Cy3 (green) or Cy5 (red), and simultaneously hybridized to an array containing spots of DNA sequences. Ratios of fluorescence intensities provide a relative measure of expression at each spot on the array.

In single channel oligonucleotide arrays, Affymetrix, short (25-mer) oligonucleotides are synthesized in situ by photolithographic methods and attached to the array. Each gene is represented by a set of spots on the array called a probe set. Unlike cDNA arrays, only a single sample can be measured on the chip at a time and two separate hybridizations are required to study differential expression.

Agilent combines two-sample hybridization with the use of long (60-mer) oligonucleotides. These arrays are also hybridized with two different fluorescent samples and measurements of differential expression obtained from the relative abundance of hybridized mRNA.

Pre-processing and normalization of microarray data, with the goal of controlling the effects of systematic error while retaining full biological variation, are critical to obtaining valid results. It is now recognized that these steps are platform specific and difficult to automate [1,2]. Two color cDNA microarrays and single color Affymetrix oligonucleotide chips have been extensively studied and platform specific recommendations for the analysis of data from these microarray technologies are well documented [3-5], for cDNA arrays, and [6] for Affymetrix arrays. Agilent microarrays have not enjoyed the same degree of scrutiny to date.

The goal of this study is to quantify some of the sources of error that affect measurement of expression using Agilent arrays and to compare Agilent's Feature Extraction software with pre-processing methods that have become the standard for normalization of cDNA arrays. These include log transformation followed by loess normalization with or without background subtraction and often a between array scale normalization procedure. The larger goal is to define best study design and pre-processing practices for Agilent arrays, and though this goal is not fully realized, we offer some early suggestions.

Agilent's Feature Extraction algorithms were developed with the aim of reducing systematic errors that arise from labeling bias, irregular feature morphologies, mismatched sample concentrations and cross-hybridization, [7]. They quantify feature signals and their background, perform background subtraction, dye normalization, and calculate feature log ratios (Agilent's Processed Signal value) and error estimates. The error estimates, based on an extensive error model and pixel level statistics calculated from the feature and background for each spot, are used to generate a p-value for each log ratio.

The file generated by Agilent's extraction software also contains raw pixel intensity data. These intensities, either mean or median values, can easily be exported to other software, such as R [8].

## Results and discussion

Three datasets, representing three mammalian species, are used in this paper. Full details and data quality statistics are described in the Methods section. To avoid duplication, most results are shown only for a few arrays. However, all three experiments yielded very similar results, as have other Agilent datasets prepared at this institution but not available for publication here.

### Human cancer cell line DU-145

The first dataset uses the human cancer cell line DU-145, treated with two doses, 2 uM and 5 uM, of 5'Aza-2'deoxycytidine. Treated RNA was labeled with Cy3 (green) and untreated RNA labeled with Cy5 (red) on two arrays. A third array is a dye-swap hybridization of the 5 uM dose.

### Murine prostate development

The second dataset is from a study of prostate development in the mouse. Total RNA was isolated and pooled from same-sex siblings for each of five age-matched litters. For each litter, competitive hybridization (male vs female) with dye swap was performed using Agilent 44 K mouse arrays.

### Canine self-self with spike-in

**Figure 1:**
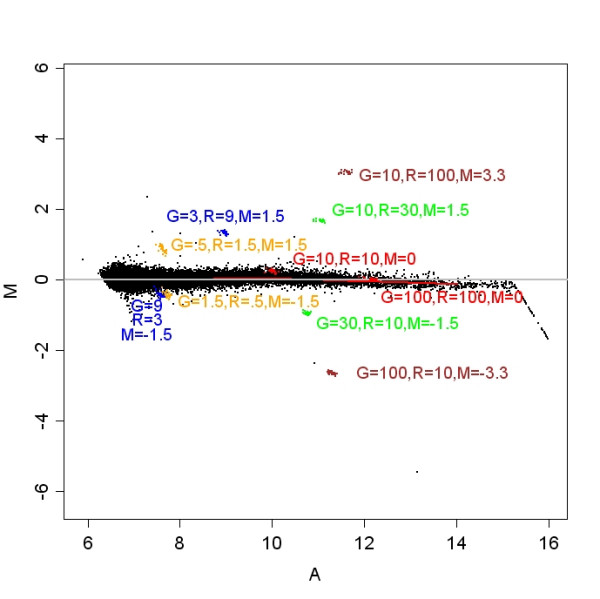
**Spike-in array**. This scatterplot shows the design of the spike-in experiment. The log fold change for each probe (M) is shown as a function of the mean single channel intensity (A). This array was pre-processed using loess normalization without background subtraction. R and G indicate spike-in concentration levels in the red and green channels. Observed fold changes reflect spike-in concentrations with the exception of the blue points in the lower left and to some extent the orange points in the same area. This array is representative of the spike-in arrays in this experiment.

For the third dataset, total RNA was isolated from one dog brain sample and applied to both channels on each of four Agilent canine 44 K arrays. RNA spike-ins were used from Agilent's two-color RNA spike-in kit following manufacturer's instructions. Table 1 summarizes the design of the spike-in experiment used in this study and Figure 1 shows an MA plot of one array. Note that the lowest intensities in Figure 1 are not represented by spike-in probes.

**Table 1:** Design of spike-in experiment. The spike-in controls are two sets of ten synthesized RNA mixtures derived from the Adenovirus E1A transcriptome [31] with different concentrations in each set. The A mix was hybridized with Cy3 and the B Mix with Cy5 on each array, according to manufacturers recommendations, and in the recommended quantity.

RNA spike-in name	A Mix relative copy number	B Mix relative copy number	expected ratio A/B
(+)E1A_r60_1	10	10	1:1
(+)E1A_r60_n11	1.5	0.5	3:1
(+)E1A_r60_a20	100	100	1:1
(+)E1A_r60_3	3	9	1:3
(+)E1A_r60_a104	10	30	1:3
(+)E1A_r60_a107	30	10	3:1
(+)E1A_r60_a135	9	3	3:1
(+)E1A_r60_a22	10	100	1:10
(+)E1A_r60_a97	0.5	1.5	1:3
(+)E1A_r60_n9	100	10	10:1

### Pre-processing and normalization

The pre-processing methods compared in this paper include Agilent's Feature Extraction and simple loess normalization, as implemented in the limma Bioconductor package [9]. Loess normalization is considered without a background correction step as well as with background subtraction. These normalizations are compared using graphical presentations, fold change estimates, empirical Bayes t-statistics and ROC curves.

**Figure 2:**
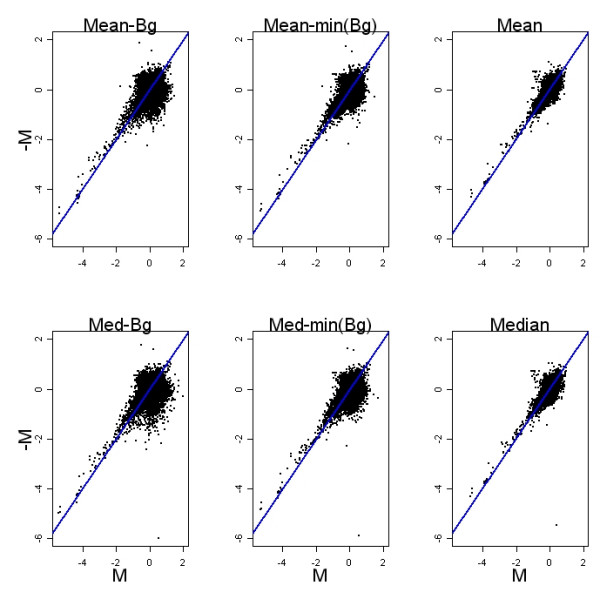
**Mean vs median dye swap plots**. Each panel shows log_2 _fold changes from the same two dye-swapped arrays. The effect of choosing mean or median to summarize spot intensity is seen by comparing across rows. Background correction methods can be compared across columns. Mean or median dye swap correlation is similar. Both pre and post-normalization, correlation from highest to lowest is no background adjustment, minimal constant adjustment and local background subtraction.

Image processing was performed using Agilent's Feature Extraction Software. After image processing, the intensity of each spot is summarized by the mean or median pixel intensity of the spot, as well as a measurement of inter-pixel variability within the spot. We found the choice of mean or median pixel intensity had little impact on downstream analysis. Both pre and post normalization, there were no marked differences in standard exploratory microarray plots using either measure. Log_2 _fold changes from a representative dye-swap pair in the prostate development study are shown in Figure 2. We chose the median for further analyses.

After image analysis, the Feature Extraction software and the marray or limma R packages in Bioconductor were used for further pre-processing. Both Bioconductor packages have functions for reading the data, plotting images, and within and between array normalizations. The limma package also implements linear modeling for selecting differentially expressed genes and has functions for alternative methods of background correction.

**Figure 3:**
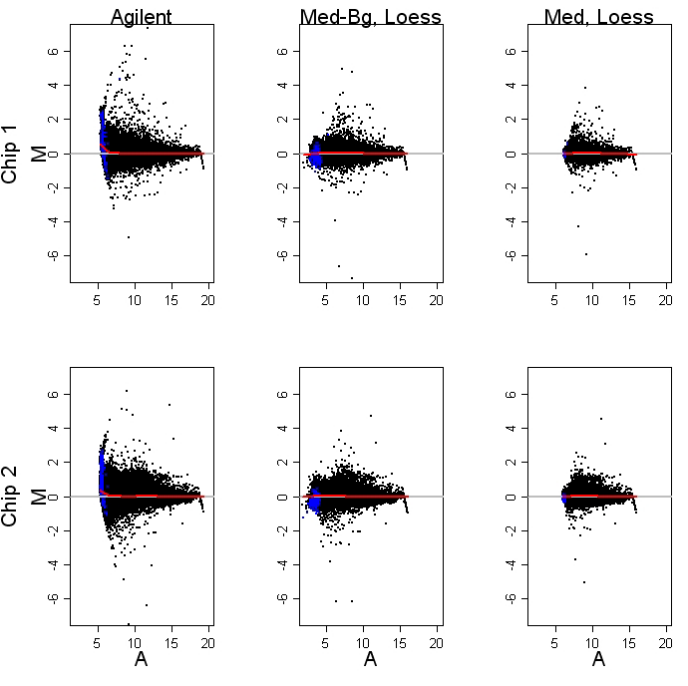
**Post-normalization MA plots**. MA plots show log fold changes (M) as a function of the mean single channel intensity (A). Columns show the effect of pre-processing methods. The two arrays shown in the rows are typical of results seen with Agilent chips. The Agilent normalized MA plot exhibits large variablity at low intensities and a low intensity bias toward positive fold changes. Background subtracted, loess normalized MA plots of median or mean fold changes are more variable than the same MA plots when background is not subtracted.

Post-normalization data for the prostate development study are shown in the MA plots in Figure 3. Loess curves calculated with positive and negative controls removed are plotted in red. The red lines give a robust profile of the mean fold change as a function of average intensity. Blue highlighted points are the negative controls which should show very little hybridization and no differential expression. The MA plot of Agilent normalized data exhibited large variablity at low intensities and a low intensity bias toward positive fold changes. This bias was also reflected in the negative controls. Background subtracted, loess normalized MA plots showed more low intensity variablity than the same MA plots when background was not subtracted. Negative control spots were more evenly scattered around the zero line than they were in the Agilent normalized plot.

**Figure 4:**
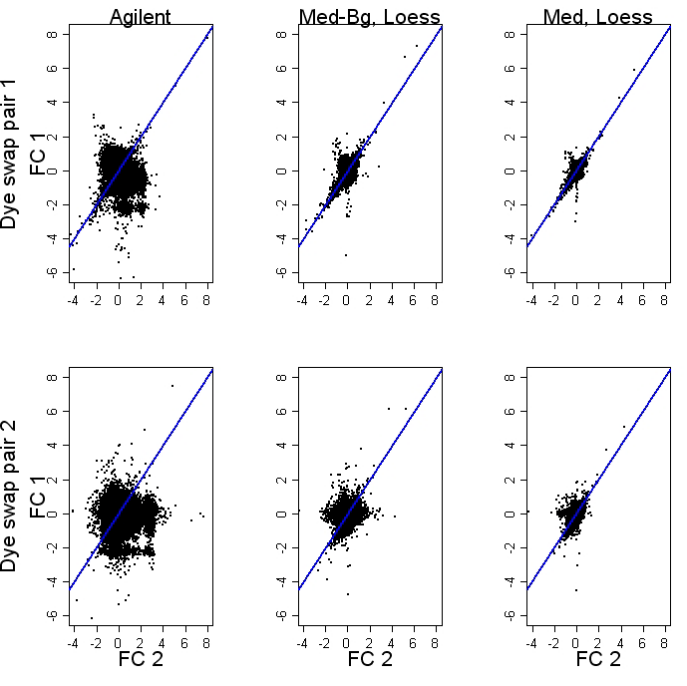
**Post-normalization dye swap plots**. Each row shows log_2 _fold changes from the same two dye-swapped arrays. Pre-processing methods are compared across columns. The arrays shown in the rows are representative of results seen with Agilent chips. Agreement across the hybridizations is best for probes that are more than two-fold differentially expressed and when no background subtraction is used.

Scatterplots of log_2 _fold changes for arrays containing biologically identical samples (technical replicates) are a useful visualization for comparison of normalization algorithms. The dye-swap design employed in the prostate development experiment is ideal for this comparison, allowing consideration of dye-related effects as well as other sources of technical error. Figure 4 shows Scatterplots of log_2 _fold changes calculated on pairs of dye-swapped arrays. Log fold changes from the dye-swapped array were inverted to show treatment/control in both dimensions. Dye-swap Pearson correlations were higher without background subtraction (range: 0.33, 0.68) than with background subtraction (range: 0.21, 0.54). The Agilent processed signal in these plots exhibited the largest variability.

**Figure 5:**
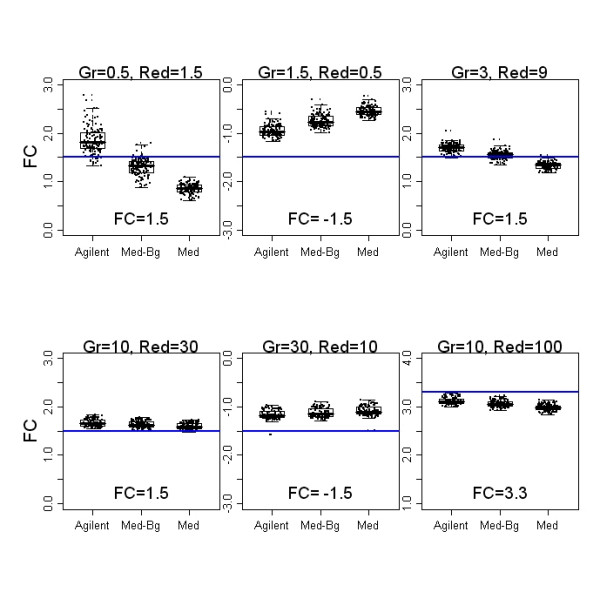
**Variance, bias and background subtraction**. Boxplots of the spike-in probes from the self-self experiment show increased variability with Agilent processing and with background subtraction. Each spiked-in probe is spotted 30 times on the array. All 30 replicates on each of the 4 arrays are individually plotted as well. Horizontal "jitter" was added to separate overlayed points. Bias, the distance from the reference line, is greatest for no background subtraction. The first two plots in the top row are lower intensity and this is where the increased variability with background subtraction is most apparent. Intensity increases for the remaining plots and the difference is minimal in the lower set of plots.

The rationale for subtracting a measure of background from the signal intensity is the assumption that the fluorescence of a spot is the sum of signal intensity and some background noise which is due to a variety of technical factors. If background is included in the estimate of the signal for a spot, then the result is a biased estimate of the true hybridization. This is particularly prominent at low signal intensities, resulting in an underestimate of the fold changes. On the other hand, subtraction of background involves an additional estimate which will increase the variability of the signal log ratios [3]. This extra variability is clearly seen in both the MA plots and the dye-swap plots. Boxplots of the spike-in probes from the canine self-self experiment, Figure 5, show the bias that results when background is not subtracted. The horizontal reference line in each plot is the target fold change for that probe. The first two plots in the top row are low intensity probes and this is where failure to subtract background has the greatest effect on fold change. As spike-in concentration and intensities increase, the effect on fold change is smaller. Note that increased variability of the Agilent pre-processing is seen in the lowest intensity spike-in features as well. For all methods, however, the range of observed fold changes did not include zero and the distributions were tight with few outliers.

To explore the effect of background subtraction further, we viewed our post loess normalized dye-swap data using three levels of background subtraction, a local background subtraction method, a minimal constant background subtraction and no background adjustment, Figure 2. The minimal constant background adjustment subtracted the minimum value of background for the entire array from each signal intensity. Both pre and post-normalization, the highest dye-swap correlation was observed with no background adjustment, followed by the minimal constant adjustment and the lowest correlation was seen with local background subtraction. This was the expected result of increasing variability by using two estimates to quantify intensity, foreground minus background, as opposed to using the foreground estimate only.

**Figure 6:**
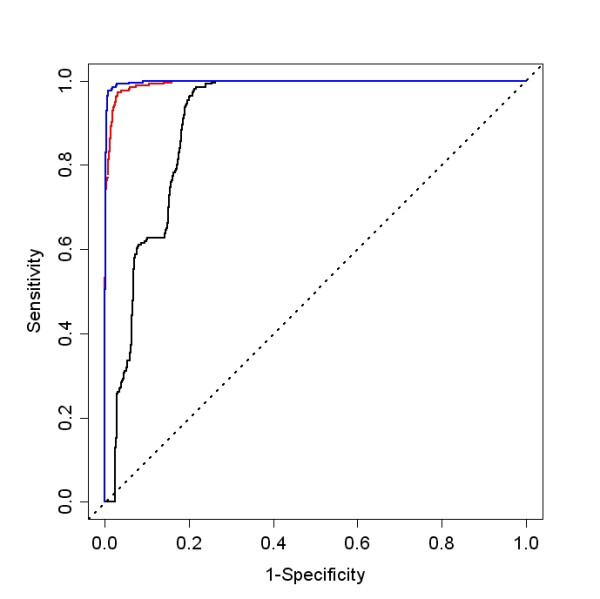
**Single array ROC curves for fold change and spike-in probes**. These ROC curves display the ability to identify differentially expressed probes based on the fold change value. In the spike-in experiment, only the spiked-in probes are present in different quantities in the two channels. The spike-in probes are identified better using median fold changes either with or without background subtraction compared to Agilent processing. Agilent, median with and median without background subtraction, black, red and blue lines respectively.

**Figure 7:**
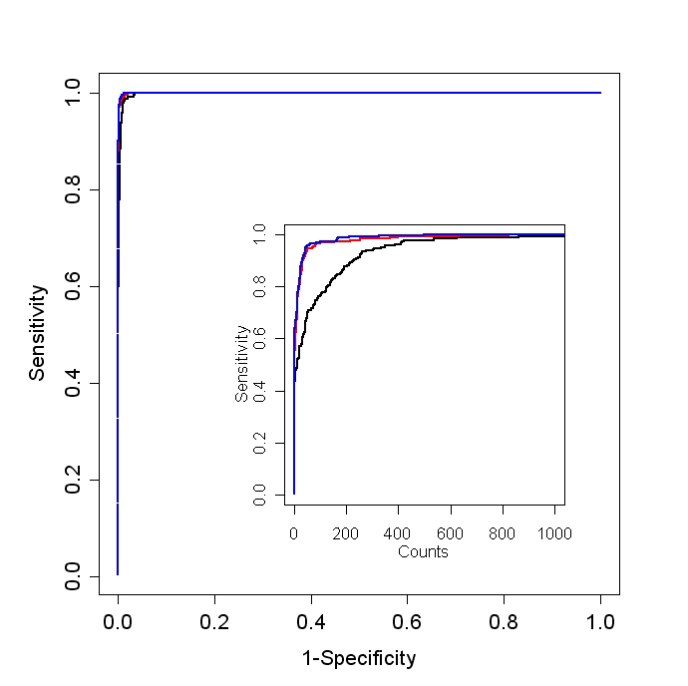
**Multiple array moderated t statistic ROC curves**. These ROC curves display the ability to identify differentially expressed probes based on the moderated t-statistic. In the spike-in experiment, only the spiked-in probes are present in different quantities in the two channels. This t-statistic takes the variance of the fold changes into account in a way that borrows strength across all genes. This is necessary with a small number of arrays to reduce the possibility that genes having extremely small variances, by chance alone, are identified as significant. Here the three pre-processing methods performed equally well. The inset of this figure is the same graph where the x axis has been truncated and 1-specificity has been replaced by counts. This shows the very minor improvement using loess normalization with or without background subtraction compared to Agilent normalization. Agilent, median with and without background subtraction, black, red and blue lines respectively.

Ultimately, the best criteria on which to base the selection of a pre-processing method is whether it gives the correct answer. The spike-in experiment, though small, does allow us to compare observed results to expected. Because the background RNA is the same in both samples, only the spiked-in genes, comprising a total of 240 spots on each array, are present in different quantities in the two channels. ROC curves were used to determine how well these spike-in probes, with expected fold changes greater than zero, could be distinguished among the set of non-spiked in probes and probes spiked-in with expected fold changes of zero. Fold changes calculated after loess normalization, with or without background subtraction, gave higher specificity for differential expression than after processing with the Agilent Feature Extraction algorithm, Figure 6. The area under the ROC curve for the Agilent processed signal was 88.8% compared to 99.4% for loess normalization with background subtraction and 99.7% without background subtraction. When a moderated t-statistic, or a z-statistic in the case of Agilent processing, calculated from the four arrays was used, the three methods performed equally well: Agilent AUC = 99.8%, median with background subtraction AUC = 99.9%, and median without background subtraction AUC = 99.9%, Figure 7. The inset of Figure 7 is the same graph where the x axis has been truncated and 1-specificity has been replaced by counts. This shows the very minor improvement using loess normalization with or without background subtraction compared to Agilent normalization. The fact that the AUCs are uniformly large indicates that the spike-in experiment does not include a sufficient number of low intensity probes to fully assess the performance of these methods.

It should be noted that, although loess normalization performs well in many cases, there are experiments for which it may not be appropriate. Loess normalization requires the assumption that either most of the genes are not differentially expressed across the range of intensities or that there are an approximately equal number of up and down regulated genes across the intensity range [2]. A spike-in experiment, having a small number of spiked-in transcripts with a symmetric design, is ideal for loess normalization [10]. If the design of an experiment does not meet these assumptions (e.g. when a disease specific array is used a large proportion of genes might be differentially expressed), an alternative normalization may be needed. The recent paper by Oshlack et al. [11] discusses normalization for boutique arrays and presents an alternative weighted loess normalization that could be considered in this case.

We considered a between array scale normalization procedure [2] but found it to add variability to the spike-in experiment used in this study. Yang et al. [12] have similarly observed that cross-array normalization can increase mean square error. Given the importance of the spike-in experiment for the evaluation of pre-processing methods, we felt that we could not provide a fair evaluation of cross-array normalization. We believe that the additional variation in the spike-in log ratios observed after this cross-array normalization may be explained by the methodology for the spike-in experiments. Specifically, spike-ins are added into the sample relatively late in the preparation of the cDNA samples, and the log ratios of the spike-in genes are therefore subject to less cross-array variability than the naturally expressed transcripts of similar abundance. As a result, cross-array normalization based on naturally expressed transcripts can paradoxically add variability to the spiked-in transcripts.

### Dye effects

Several investigators [13-16] have observed that gene specific dye effects persist in two-color arrays after global normalization procedures. Some have recommended that experiments incorporate dye-swaps, common reference designs, or other methods for controlling dye effect, while others argue that this is unnecessary, [14] and [17]. The self-self design of our spike-in experiment permits an investigation of the number and magnitude of uncorrected dye effects.

**Figure 8:**
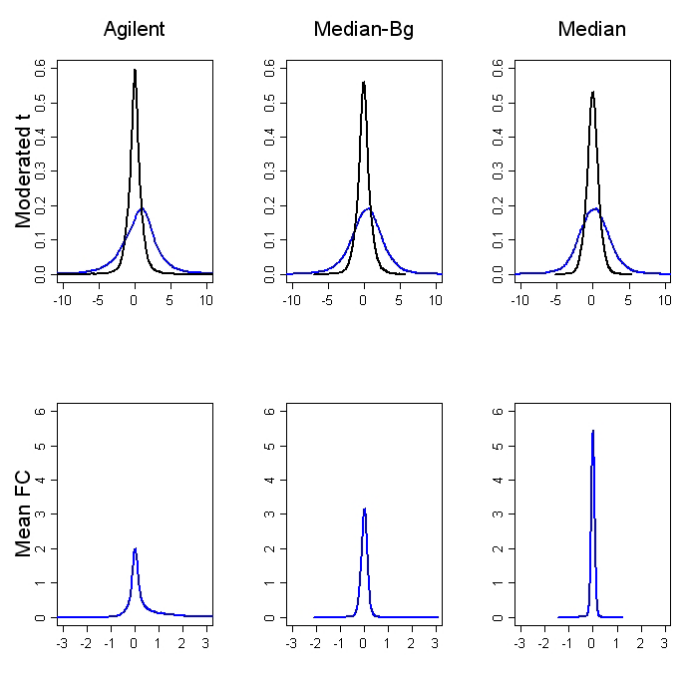
**Dye effects: non spike-in probes**. This figure shows the distribution of dye effects. The actual distributions are shown in blue and null distributions are shown in black. Pre-processing methods are compared across columns. In the top row moderated t-statistics are used to measure dye effect. The null distribution was obtained by randomly changing the sign of half of the log ratios for each gene before calculating the t-statistic. Gene specific dye effects are indicated by the heavy tails on the blue curves compared to the black curves of the null distribution. There is also a slight shift to the right for the Agilent processed data and the loess normalized, background subtracted data. In the bottom row, observed distributions of dye effects, as measured by the mean fold changes, are shown to give a sense of their scale. The relationship between null and observed distributions is similar to that seen with moderated t-statistics and is not shown here. Dye effects tend to be small regardless of the pre-processing method used.

We investigated the presence of dye effects using one-class moderated empirical Bayes t-statistics from the non spiked-in probes in the self-self experiment. The observed and null distributions are compared in Figure 8. The null distribution was obtained by changing the sign of two of the four log ratios for each gene so that these two represent Cy3/Cy5 instead of Cy5/Cy3. Moderated t-statistics for each normalization algorithm are shown in the top row of Figure 8. In both background corrected normalizations, the peak of the observed curve is shifted in the positive direction, and in all three plots, the observed distributions have heavier tails than the expected. These results suggest that gene specific dye effects do remain following normalization and that background subtraction, which amplifies the noise at the low end of the intensities, may also amplify some of these dye effects. If these amplifications are more positive than negative, this would explain the shift in the observed distributions of the moderated t-statistics when background is subtracted. The moderated t-statistics for dye effect are largest after Agilent processing and smallest after loess normalization without background subtraction. In the bottom row of this figure, observed distributions of the mean fold changes for each pre-processing method give an idea of the magnitude of the raw dye effects. It is encouraging that the effects tend to be quite small for the vast majority of genes, regardless of the processing method used.

An estimate of the number of genes affected by dye bias was also calculated from the self-self experiment. If a feature has no remaining dye effects after normalization, then in a self-self hybridization, the log ratio log_2_(*R/G*), measured on an arbitrary array, is equally likely to be positive or negative. Thus, the number of positive log ratios observed over *n *arrays will have a *null *binomial distribution with probability of success *p *= 0.5.

The distribution observed in our self-self experiment, Table 2, has a distinctly heavier tail than does the null distribution, consistent with the previous observation that dye effects linger after normalization. In order to estimate the number of affected spots, a three component binomial mixture model was fit to the four arrays in the self-self experiment:

**Table 2:** Number of genes with dye effects. Observed, expected and best fitting mixture distributions of the number of positive log ratios. Observed distribution in the self-self experiment has distinctly heavier tails than the expected binomial null distribution.

pos. log ratios	0	1	2	3	4
observed dist	0.176	0.207	0.236	0.205	0.177
binomial (p = 0.5)	0.062	0.250	0.375	0.250	0.062
best fitting mixture	0.177	0.206	0.235	0.206	0.177

π2binom(p,4)+(1−π)binom(0.5,4)+π2binom(1−p,4).
 MathType@MTEF@5@5@+=feaafiart1ev1aaatCvAUfKttLearuWrP9MDH5MBPbIqV92AaeXatLxBI9gBaebbnrfifHhDYfgasaacH8akY=wiFfYdH8Gipec8Eeeu0xXdbba9frFj0=OqFfea0dXdd9vqai=hGuQ8kuc9pgc9s8qqaq=dirpe0xb9q8qiLsFr0=vr0=vr0dc8meaabaqaciaacaGaaeqabaqabeGadaaakeaadaWcaaqaaGGaciab=b8aWbqaaiabikdaYaaacqqGIbGycqqGPbqAcqqGUbGBcqqGVbWBcqqGTbqBcqGGOaakcqWGWbaCcqGGSaalcqaI0aancqGGPaqkcqGHRaWkcqGGOaakcqaIXaqmcqGHsislcqWFapaCcqGGPaqkcqqGIbGycqqGPbqAcqqGUbGBcqqGVbWBcqqGTbqBcqGGOaakcqaIWaamcqGGUaGlcqaI1aqncqGGSaalcqaI0aancqGGPaqkcqGHRaWkdaWcaaqaaiab=b8aWbqaaiabikdaYaaacqqGIbGycqqGPbqAcqqGUbGBcqqGVbWBcqqGTbqBcqGGOaakcqaIXaqmcqGHsislcqWGWbaCcqGGSaalcqaI0aancqGGPaqkcqGGUaGlaaa@6093@

The choice of three components reflects both the symmetry observed in the data and the small number of arrays. The center component represents the null case, and its mixture coefficient 1 - *π *estimates the proportion of array features without dye effect. Features with dye effect are segregated into the two flanking components. By taking advantage of the symmetry evident in the observed distribution, this model requires only two free parameters: *π*, representing the proportion of genes with dye effect; and *p*, which for our purposes is a nuisance parameter. The model was fit on a grid of parameter values, and the optimal fit was obtained when *p *= 0.1 and *π *= 0.43. It is of concern that more than 40% of genes exhibited a persistent dye effect, even though these effects were small. A 99% confidence interval of (0.39, 0.47) for the proportion of genes with dye effect was estimated by parametric bootstrap, where the bootstrap was taken over genes rather than samples. The corresponding values of the nuisance parameter,  *p*, varied between 0.08 and 0.12. Details of the algorithm are given in the methods section.

### Error model

Measurement errors in a microarray experiment are a combination of systematic and random components that can arise at any step during the study: array manufacture, mRNA preparation, hybridization, scanning, and imaging. Background subtraction and dye normalization are techniques used to adjust raw data for known systematic errors, while error modeling estimates the additional variability in a spot due to random error or any unknown systematic errors.

Agilent's default method for estimating within spot variability is a hybrid. Under this model, the error estimate used for each spot is the larger of a universal error model and a propagated error model. For the propagated error model, estimates of random error for individual log fold changes are based on pixel level statistics and propagated through the background subtraction and normalization steps to the final log ratio. The universal error estimate is calculated using additive and multiplicative adjustment terms that have been estimated with many platform specific self-self experiments. It represents the expected error of the difference between the red and green channels [18]. On these arrays the universal error model produced the larger estimate of error for > 99.9% of the probes. Based on these error estimates, z-statistics and p-values are calculated for each probe.

**Figure 9:**
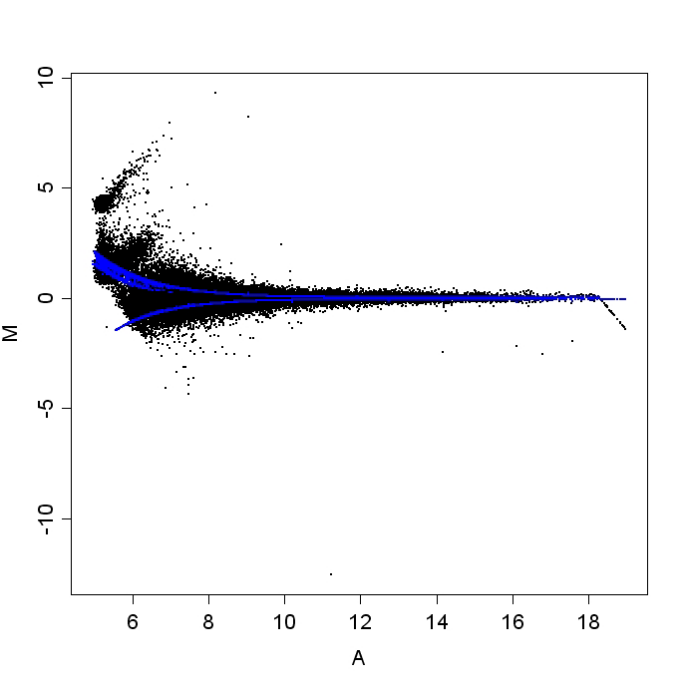
**Agilent error model**. This figure illustrates Agilent's universal error model. The scatterplot shows log fold changes (M) as a function of the mean single channel intensity (A) for a single Agilent processed array. Every log fold change (black point) is matched with a blue point of the same mean intensity level which shows the error in the log fold change as estimated by Agilent's universal error model. For log fold changes that were negative, the error estimate was multiplied by -1 before plotting. These error estimates capture the large global variation that characterizes low intensity genes after Agilent pre-processing. This array is representative of results seen with Agilent arrays.

An Agilent processed MA plot from the spike-in study is shown in Figure 9. Every log fold change (black point) is matched by a blue point with the same A value which shows the error in the log fold change, estimated according to the Agilent universal error model. When log fold changes are negative, errors are multiplied by -1 before plotting to better illustrate the pattern. The pattern of blue dots shows that while the error model is not a simple function of A alone, it can be fairly well approximated by such a function. The model clearly captures the large variation that characterizes low intensity genes after Agilent pre-processing. When ROC curves were drawn using the z-statistics that Agilent provides, the area under the curve improved from the 89% observed for unstandardized log ratios to 99%. Thus, the performance of the z-statistics was similar to that of fold changes calculated after loess normalization.

**Figure 10:**
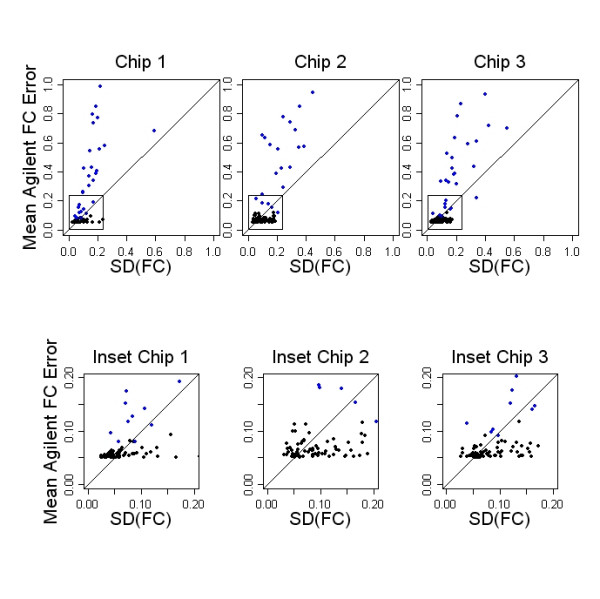
**Accuracy of Agilent universal error model**. As part of the Agilent chip design, a set of 100 oligonuceotide sequences are each represented on the array by 10 separate spots. These are useful for evaluating errors in the estimation of intensity. The vertical axis of each panel shows the mean error, measured across replicate spots, using Agilent's universal error model. The horizontal axis shows the observed standard deviation for the same replicates. Each column shows results for a single array. Some probe to probe differences are captured with the Agilent error model for low intensity probes (blue spots). The bottom row gives a close-up of the high intensity end of each array. For high intensity probes (black), where the level of error is small, the universal error model also predicts low error, however, probe to probe differences are not correlated. These arrays are representative of results seen with Agilent arrays.

The estimates provided by the universal error model closely follow the general contours of the MA plot, and we believe that it captures sources of systematic variation introduced in pre-processing. It is possible that the universal error model also captures probe specific variability. To determine how well the model performs for individual probes, we considered 100 probes that are each represented on the Agilent Human 22 K array by 10 separate spots. Figure 10 shows the three arrays from the human prostate cancer dataset. Each point represents one of the 100 replicated sequences. The observed standard deviation of the 10 replicate spots is plotted on the horizontal axis against the mean Agilent error model estimate for the 10 replicate spots, shown on the vertical axis. Pearson correlation coefficients are 0.41, 0.55 and 0.30 for the three arrays. Low intensity spots, those having mean single channel intensity less than 200, are shown in blue. For these probes, where A values are useful predictors of error, some of the sequence specific differences in errors are captured with the Agilent error model, although approximation to the actual error is poor. For higher intensity spots (black), where observed error is small, the universal error model also predicts low error, but these model based estimates do not show appreciable association with the estimates made from the observed data. Our results are consistent with the hypothesis that the error model primarily captures systematic variation introduced in pre-processing, and there is no compelling evidence to support the alternative hypothesis that additional probe specific variability is captured by the error model. However, these results are not conclusive, and a much larger set of replicated probes would be necessary to fully characterize the variability in the error estimates and evaluate the competing hypotheses.

## Conclusion

Although it has become one of the leading expression array technologies, Agilent arrays have not enjoyed the intense scrutiny that has led to the development of very effective processing methods for some competing platforms. The objective of this study was to evaluate sources of error and, specifically, to understand dye effects in the Agilent platform with a consideration for the equilibrium between budgetary supply and data demand that is so crucial to study design decisions.

We came to prefer a simple pre-processing procedure that consists of a log transformation of the intensities followed by an intensity-dependent loess normalization applied to each array. This is consistent with the current practices for cDNA arrays which were developed as a result of careful analysis of the technology and extensive experimentation [10,19-22]. On the matter of background subtraction, we prefer no background subtraction, but appreciate the arguments on both sides. Two forces drive our preference for no background subtraction. The first, entirely philosophical, is an aversion to unnecessary data processing. The second is provided by the analyses presented here, which we believe demonstrate a perhaps slight but nonetheless clear advantage for this pre-processing method.

As Figure 5 shows, Agilent's feature extraction algorithm does result in less biased fold changes, particularly for low abundance transcripts, but a great deal of variability is introduced, especially at the low intensity end. The ROC analysis makes it clear that the ability to detect differentially expressed genes suffers as a result. Either of the simpler pre-processing methods is a better choice.

It is difficult to tease out the components of the Feature Extraction Algorithm that contribute the excess variability seen in Figures 2-4. However, based on our analysis of background subtraction with loess normalization, it may be surmised that background subtraction plays a role here as well. Another pre-processing step that may contribute to the variability is the use of a surrogate intensity value when the measured intensity of an array feature is not significantly different from background levels. This step is applied separately in the two channels so that for example, the intensity measured on the Cy5 channel might just meet criteria, while the similar value measured in Cy3 just misses it and is replaced by a generic surrogate value.

The question of whether or not to subtract background is a difficult one. The fold changes for low intensity genes are clearly attenuated toward zero when background is not subtracted. On the other hand, the additional background estimation error at the low intensity end might overwhelm signal, even without fold change attenuation. We believe that making present/absent calls does not help to resolve the dilemma. Those transcripts that are expressed at the lowest levels can not be distinguished from noise and present/absent calls essentially foreclose on these. These transcripts are always difficult to find and one should not make it impossible to detect them.

Rather than considering the relative merits and risks of bias and variance separately, we prefer to evaluate the contributions each makes to mean squared error, leaving a more accurate quantification for follow up work in the lab. The data presented here are not amenable to evaluating mean squared error reliably. However, Scharpf et al. [23] have used simulations in an extensive study of the issue. These simulations show that in most cases, the largest portion of mean squared error for low intensity genes is due to random background estimation error rather than the systematic shrinkage of fold changes. It was found that this is true unless there is clear correlation between background and foreground, indicating that local background is an important component of foreground intensities. In the arrays used here, a dozen or so outlying spots on each array (data not shown) drive correlations of 0.00–0.06 between foreground and background, but in general no relationship is evident.

The noise evident in dye-swap plots and the slight advantage that ROC analysis shows for non-subtracted intensities, together with Scharpf's findings, lead us to vote against background subtraction. It should be noted, however, that when error was taken into account, in t-statistics or Agilent's z-statistics derived using the universal error model, all methods gave near perfect results in this study. The inclusion of additional spiked-in probes at lower intensities would permit a larger and more sensitive experiment. We second the recent recommendation of Tong et al. [24] that additional controls, suitable for use with spiked-in RNAs, be included on arrays.

Several datasets, including Human, Mouse and Dog samples, yield similar results giving confidence that results can be generalized. In addition to the specific datasets included in this publication, the conclusions reflect the consensus of a larger institutional experience.

At this time, the microarray community agrees that with fixed budgets and competing scientific needs, biological replication is more important than technical replication in expression studies. However, gene specific dye effects, unaddressed by within array normalization procedures, are potentially a significant source of error. Therefore, for two color arrays, dye-swap replicates are often included in study design to control this source of error.

In our Agilent spike-in study, gene specific dye biases persisted after normalization, affecting nearly half the genes. For all but a few genes, however, these effects were quite small. Because many genes were involved, we suggest incorporating dye-swaps when budget and experimental design permit. Often it is possible to fold a dye swap into existing biological replicates, hybridizing treatment with Cy5 in one sample and with Cy3 in another. Because dye-swap plots after loess normalization without background subtraction show substantial agreement and because gene specific dye effects were generally small, we prefer not to spend arrays on dedicated dye-swaps.

## Methods

### Data

Natural experimental datasets as well as data from an artificial spike-in experiment were used in this study to evaluate pre-processing methods. Spike-in experiments have been used extensively to study pre-processing methods [25-30]. Although such experiments usually only look at a small number of spiked-in transcripts from foreign species, they have been crucial to recent advances in the field, allowing investigators to objectively compare estimated measures of expression to relative transcript abundance. The recent study by Tong et al [24] investigated the use of foreign spike-in controls in a variety of platforms including Agilent, recommending that they be routinely used in quality control checks and calling for the inclusion of many more such probes on commercial arrays. That study did not compare pre-processing methods for Agilent arrays but found, as this study did, that Agilent pre-processing produced accurate measurements of fold changes for the spiked-in transcripts. Because spike-in experiments are so highly controlled, they do not necessarily approximate natural experimental conditions. Therefore, two natural experimental datasets were included in this study to permit a more thorough characterization of the costs and benefits of each pre-processing method.

### Human cancer cell line DU-145

The human cancer cell line DU-145 was treated with two doses, 2 uM and 5 uM, of 5'Aza-2'deoxycytidine daily for four days and cells harvested on the fifth day. Three 22 K Agilent Human 1A (V2) oligonucleotide chips were used. Treated RNA was labeled with Cy3 (green) and untreated RNA labeled with Cy5 (red) on two arrays, one for each dose. For the third array, a dye-swap hybridization of the 5 uM dose, the dye assignment was reversed and treated RNA was labeled with Cy5 and untreated cells were labeled with Cy3.

### Murine prostate development

This study investigated sexually dimorphic differentiation of the embryonic prostate rudiment, the urogenital sinus (UGS). Total RNA was isolated and pooled from same-sex sibling UGSs for each of five age-matched litters. For each litter, competitive hybridization (male vs female) with dye swap was performed using Agilent 44 K mouse arrays.

### Canine self-self with spike-in

Total RNA was isolated from one dog brain sample and applied to four Agilent canine 44 K arrays. RNA spike-ins were used from Agilent's two-color RNA spike-in kit following manufacturer's instructions. The spike-in controls were two sets of ten synthesized RNA mixtures derived from the Adenovirus E1A transcriptome [31] with different concentrations in each set. These spike-ins sets were mixed with the two samples and co-hybridized to an array. Observed and expected log ratios for the thirty duplicated probes for each of the ten spike-in concentrations were then compared. Concentrations are summarized in Table 1, and in Figure 1.

### Data preparation and quality

Using Agilent's recommended protocol, hybridizations were mixed with equal amounts of labeled nucleic acid instead of equal amounts of labeled Cy dyes. The ratio of the amount of labeled Cy dye to nucleic acid (pmol/ug) was monitored and in an acceptable range. The ideal scan is one in which the same amount of red and green signal is acquired in each channel, resulting in a pixel ratio of approximately 1.0. In many studies maximum detection is preferred and in this case the Agilent default PMT (photo-multiplier tube) setting of 100% for both red and green channels was used to maximize sensitivity of detection. This was followed by post-acquisition normalization to correct for variations in relative signal intensities. The resolution setting for scanning can be either 5 or 10 micrometers. For the 22 K and 44 K arrays used in this study the spot sizes are relatively large, 135 micrometers in diameter, and therefore the 10 micrometer resolution setting was used. The method used to define spots and measure signal was the CookieCutter method described in the Agilent manual with the cookie percentage set to 0.65.

The percentage of features saturated (intensities greater than 65,502), in either channel for any of the arrays was less than 0.16%. Spots in which the inter pixel variance lies outside of an estimated interval, based on known Agilent noise characteristics, are flagged for non-uniformity. The percentages of any color Agilent feature non-uniform outliers ranged from 0.002% to 4.7%. The Agilent recommended feature non-uniformity outlier threshold is 5.0%.

Image plots (Spike-in data and prostate data) of foreground, background and pre-normalization log-ratios (M) were checked for spatial patterns that would indicate technological artifacts. Foreground images of the spike-in data appeared uniform, while the background images showed some spatial artifacts on the lower portion of two of the arrays. For the prostate study, background images showed only a minor scratch on the lowest dose array and the foreground images appeared uniform. There were no areas with a predominance of fold changes (M) in one direction or the other when viewed with all fold changes or when highlighting only those spots with the highest and lowest log-ratios.

Agilent arrays include four rows of negative control probes designed to show very little hybridization to a wide variety of targets from many sources. The red and green median intensity values and fold changes from these probes were plotted to find the level of background in all signals due to non-specific hybridization and to check for any spatial patterns in the background. Green intensities were always higher than red and any spatial trends were very minor.

MA plots of the spike-in data demonstrated that most of the spiked-in probes were measured well, with one exception, Figure 1, the probe with fold change of -1.5.

### Computational methods

Agilent's Feature Extraction Software was used for array image analysis and the calculation of spot intensity measurements which are considered to be *raw data *for the purposes of this study.

Before loess normalization with and without background subtraction, spot intensities were transformed to the log_2 _scale. A local estimate of background, specific to each spot and measured outside of and immediately adjacent to the spot, was used for background subtraction. Only Figure 2 depicts results using other measures of background.

Empirical Bayes moderated t-statistics [32], as implemented in the limma Bioconductor package [9], were used wherever t-statistics were required.

Agilent Feature Processing for the experiments reported in this paper used the Feature Extraction Version 7.5.1 default settings recommended by Agilent. The SpotAnalyzer and the PolyOutlierFlagger algorithms define the feature areas and flag outliers. Spots are flagged if pixel variation is too high or if the spot intensity is found to be an outlier. The BGSub, DyeNorm and Ratio algorithms perform back-ground subtraction, dye normalization and log ratio calculations. These settings result in processed signal intensities which are calculated using a spatial detrend value for background subtraction, a combination of linear and lowess dye normalization, and the hybrid error model options. The hybrid error model is a conservative setting which automatically chooses the larger error from two models: a propagated error model, or a universal error model for calculating the final error estimates. Log ratios and log ratio errors are calculated and combined to form z-statistics and probe level p-values.

### Estimation of proportion of genes exhibiting dye effects

In preparation, we eliminated all control features, including those features corresponding to the spiked-in transcripts, and centered each array to have a median fold change of 0, so that the null value of *p *would be exactly 0.5. For each array feature, we counted the number of arrays, out of four, in which the centered fold change was positive and calculated the empirical distribution. Medians were quite close to zero to start with, and the conclusion that a substantial portion of genes show dye effect does not depend on centering. The mixture model was optimized over values of *π *in the set {0.25, 0.26,..., 0.75} and *p *in {0.01, 0.02,..., 0.50}. Model fit was measured by the Kolmogorov-Smirnov distance between the vector of observed frequencies and the expected frequencies corresponding to each set of parameter values. A parametric bootstrap was used to generate a confidence interval for the mixture coefficient *π *and the nuisance parameter *p*. Specifically, at each bootstrap iteration, we sampled from the binomial mixture model with parameters set to the fitted values of *π *= 0.43 and *p *= 0.1, using a sample size of 42000 each time to match the number of genes on the array. We calculated the empirical distribution at each iteration and measured the Kolmogorov-Smirnov (K-S) distance between the empirical and expected distributions. The 99^*th *^percentile of statistic, calculated over 1000 iterations, was 0.0062, so we include in 99% confidence intervals those combinations of *π *and *p *for which the expected distribution was within 0.0062 of the observed distribution, measured by K-S distance.

## Authors' contributions

MZ participated in the design of the study, performed statistical analyses, and drafted the manuscript. GP and LC conceived the study. GP also participated in the design and provided direction throughout. WY performed the spike-in study, supervised the cell line and prostate studies and provided Agilent expertise. RS participated in the design of the study and contributed to background subtraction content. DB and ES provided the prostate mouse study and SS provided the Human cell line study. In addition to study conception, LC also participated in the design of the study, finalized the organization and content of the paper and contributed to the drafting of the final manuscript. All authors contributed to, read and approved the final manuscript.
